# Optimization of the Thickness Ratio and Roll-Bonding Parameters of Bimetallic Ti/Al Rod for Bending-Dominated Negative Thermal Expansion Metamaterials

**DOI:** 10.3390/ma17235738

**Published:** 2024-11-23

**Authors:** Feiyin Li, Sicong Liu, Shaojie Ma, Xinping Zhang

**Affiliations:** 1 School of Mechanical Engineering, Nanjing University of Science and Technology, Nanjing 210094, China; feiyinli@njust.edu.cn; 2 School of Material Science and Engineering, Nanjing University of Science and Technology, Nanjing 210094, China; l_xiaos156@163.com

**Keywords:** negative thermal expansion, metamaterials, roll-bonding, isotropy, cyclic thermal stability, titanium/aluminum composite

## Abstract

Roll-bonding has rarely been applied to prepare rods for negative thermal expansion metamaterials (NTEMs). Parameters for quantitatively assessing the isotropy and cyclic thermal stability of the thermal expansion coefficient α of NTEMs are lacking. Here, the Ti-to-Al thickness ratio in bimetallic rods for “cross-shaped” node bending-dominated NTEMs was optimized using a general model proposed in the literature. The finite element method was used to determine the optimal initial thickness ratio of the billet, as well as the reduction ratio and rolling temperature. NTEMs were prepared with roll-bonded Ti/Al rods and Ti nodes. A relatively high thermal expansion coefficient was obtained when the thickness ratio of the 7075 Al alloy of the rods was in the range of 0.56–0.60. The optimized roll-bonding process to meet this thickness ratio was as follows: a rolling temperature of 400 °C, a reduction ratio of 50%, and TA1 Ti and 7075 Al billet thicknesses of 0.5 mm and 1.5 mm, respectively. The isotropy and cyclic thermal stability ratios were proposed to quantitatively assess the isotropy and cyclic thermal stability of the NTEMs. These results help to expand the preparation and evaluation methods for NTEMs.

## 1. Introduction

Roll-bonding has found extensive applications in the production of sheets and large composite layers [1]. There are numerous research results on optimizing parameters such as reduction ratio [2], rolling temperature [3], pre-rolling surface treatment [4], and post-rolling heat treatment [5] to improve the mechanical properties and interfacial bonding strength of rolled composite materials. However, there has been little research on optimizing the rolling process to achieve the specified thickness ratios of each layer after rolling.

Negative thermal expansion metamaterials (NTEMs) have been investigated for use in aerospace vehicles and naval ships because they can maintain their thermal stability and reduce thermal stresses. NTEMs include two structures: bending-dominated and stretching-dominated structures [6]. The negative thermal expansion (NTE) of bending-dominated NTEMs occurs due to incompatible thermal deformation caused by the different thermal expansion coefficients *α* of the two materials in bimaterial rods [7]. Bimaterial rods are the most important components of bending-dominated NTEMs [8]. The processing methods for NTEMs mainly include gluing [9], welding [10], additive manufacturing [11,12,13,14], and mechanical splicing [15]. Difficult machining and uneven stress distributions in components have not yet been solved for welding, additive manufacturing, and mechanical splicing methods [16,17,18,19,20]. One of the most common industrial methods for combining dissimilar metals is roll-bonding because it is highly productive, low in cost, and suitable for mass production [21,22]. However, it has not been used to roll-bond bimetallic rods for NTEMs.

It is necessary to optimize the bimetallic rod thickness ratio in bending-dominated NTEMs to obtain a highly negative *α* [23]. The thickness ratio of bimetallic rods before rolling is different after rolling due to the different mechanical properties of the constituent metals, as well as the rolling parameters and the initial thickness ratio of the billets [5,24]. Therefore, it is necessary to optimize the bimetallic thickness ratio in the billet before rolling and then optimize the rolling temperature and reduction ratio according to the optimized bimetallic thickness ratio.

NTE isotropy or anisotropy is a popular research topic for NTEMs and has been investigated by many researchers [25,26,27]. The NTE isotropy of NTEMs is determined by comparing their deformation in orthogonal directions. For example, when the lengths (mm) in the horizontal and longitudinal direction were shortened from 190.54/184.57 to 184.70/178.95 and from 188.23/188.37 to 182.38/182.17, the NTE was shown to be isotropic [9]. Similarly, when the lengths (mm) in the horizontal and longitudinal direction were shortened from 11.198 to 10.830 and 11.060 to 10.706, the NTE was also isotropic [28]. However, a comparison of the isotropy of NTEMS is not possible due to the lack of appropriate parameters.

A high cyclic thermal stability (TCS) of *α* ensures that NTEMs will perform stably under cyclic thermal loading. The thermal stresses and damage to NTEMs generated by cyclic thermal loading are different from those generated by a single thermal load, as are the fatigue and tensile properties. Klein et al. proposed a thermomechanical model of a tetrahedral unit cell with three degrees of freedom subjected to cyclic thermal loading [29]. There are few theoretical analyses and experimental results on the *α* of NTEMs during cyclic thermal loading. Therefore, the stability of the thermal expansion properties of NTEMs under cyclic thermal loading must be further explored.

This work explored the possibility of using rolled bimetallic rods for NTEMs and proposed parameters to quantitatively assess the isotropy and TCS of *α* of the metamaterials. The thickness ratio of TA1 Ti and 7075 Al alloy rods in bending-dominated NTEMs with cross-shaped nodes was optimized based on a model proposed in the literature [23] to achieve a large negative *α*. The initial thickness ratio of the billet, as well as the reduction ratio and rolling temperature, were optimized using the finite element method (FEM) to achieve the optimized thickness ratio. The rolled rods were annealed to further improve their bonding strength. TA1 Ti/7075 Al bimetallic rods were prepared as bending-dominated NTEMs with cross-shaped nodes by the gluing method, and their thermal expansion properties were characterized. The isotropy ratio *IR* and cyclic thermal stability ratio *SR* were proposed to quantitatively assess the isotropy and TCS of *α*. These works help to expand the preparation and evaluation methods for NTEMs.

## 2. Material and Methods

### 2.1. Materials

Titanium alloys and aluminum alloys have a high specific strength and corrosion resistance and are widely used in aerospace applications. Preparing NTEMs with titanium/aluminum alloy bimetallic rods ensures that they will be lightweight, high-strength, and corrosion-resistant under working conditions. Therefore, we used commercial TA1 pure Ti and 7075 aluminum alloys. The chemical composition (wt.%) of TA1 Ti and 7075 Al alloy were measured using an elemental analyzer (Thermo Fisher Scientific, Waltham, MA, USA, FLASHSMART) and a scanning electron microscope (HITACHI, Tokyo, Japan SUS1510) with an energy-dispersive X-ray spectroscopy function, and the results are listed in Table 1.

### 2.2. Rolling and Heat Treatment

TA1 Ti/7075 Al bimetallic rods were subjected to single-pass roll bonding. The dimensions (mm) of the 7075 Al alloy and TA1 Ti billets were 100 × 100 × *t*_1_ and 100 × 100 × *t*_2_, respectively. The billets were vacuum annealed at 650 °C for the TA1 Ti alloy and 410 °C for the 7075 Al alloy. The billets were then ground to remove the surface oxide layer before rolling and riveted together. *t*_1_ and *t*_2_, rolling temperature, and reduction ratio for single-pass rolling are optimized in Section 3.2.

The rolled plates were annealed at 400 °C, 450 °C, and 480 °C for 2 h and 4 h.

### 2.3. Microstructure and Properties Characterization

The microstructure and elemental distribution of the Ti/Al interface were characterized by a scanning electron microscope (SEM) equipped with energy-dispersive X-ray spectrometry (EDS; HITACHI SUS1510).

During temperature changes, thermal stress will be generated at the bimetallic interface of NTEMs due to the different *α* values of the bimetals. When the bonding strength of the bimetals is lower than this thermal stress, the interface will crack, leading to the failure of the NTEMs. Therefore, a sufficient bonding strength is required. Plate-type specimens (10 mm wide) were prepared for the T-peel test (ASTM D1876-08) [30] performed at a deformation rate of 1 mm/min to obtain the bonding strength. Three specimens were used for each condition.

$$\mathrm{Bonding}\;\mathrm{strength}\;\sigma_{T}\;=\;\mathrm{Average}\mathrm{peel}\mathrm{force/Width}\mathrm{of}\mathrm{the}\mathrm{sample}$$

**Figure 1:**
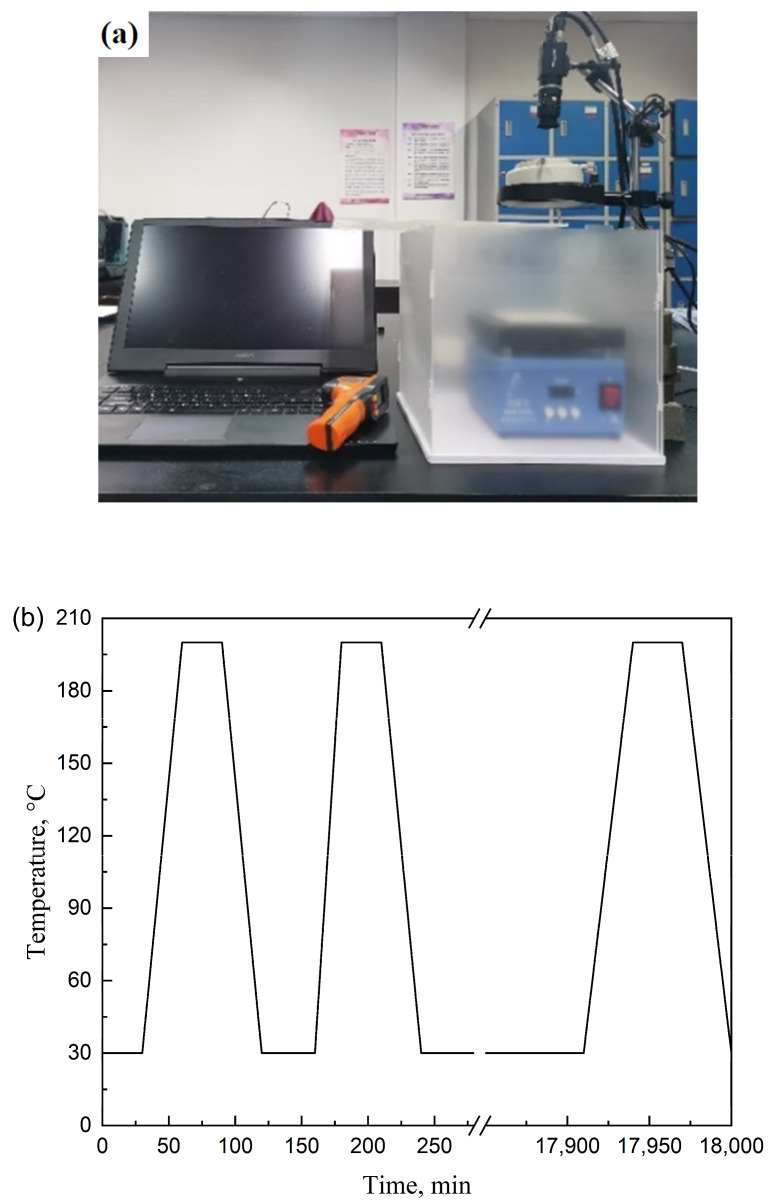
Thermal expansion test system and heating curve for cyclic thermal stability test. (**a**) Test system, and (**b**) heating curve.

The thermal deformation of TA1 Ti, 7075 Al, and NTEMs was recorded to calculate *α* using the digital image correlation method. The thermal expansion test system included a heating module, image capture module, and data processing module (DIC software, Vic2D-6), as shown in Figure 1. Before the test, these plates and NTEMs were sprayed with high-temperature resistant matte white paint and black paint and then left to air dry. The *α* of TA1 and 7075 Al with dimensions (mm) of 10 × 10 × 1.5 were measured from 20 °C to 200 °C over a period of 30 min, and *α* was determined to be 1.154 × 10^−5^/K and 2.253 × 10^−5^/K, respectively. Thermal expansion of the NTEMs in the horizontal (*X*) and longitudinal directions (*Y*) (Figure 1) was measured to determine the isotropy. Thermal expansion of the NTEMs was measured for 120 cycles to characterize the TCS. NTEMs were left at 30 °C for 30 min, then heated from 30 °C to 200 °C over a period of 30 min, held at 200 °C for 30 min, and then cooled from 200 °C to 30 °C over a period of 30 min. The interval between each cycle was 30 min.

### 2.4. Preparation of “Cross-Shaped” Node Bending-Dominated NTEMs

**Figure 2:**
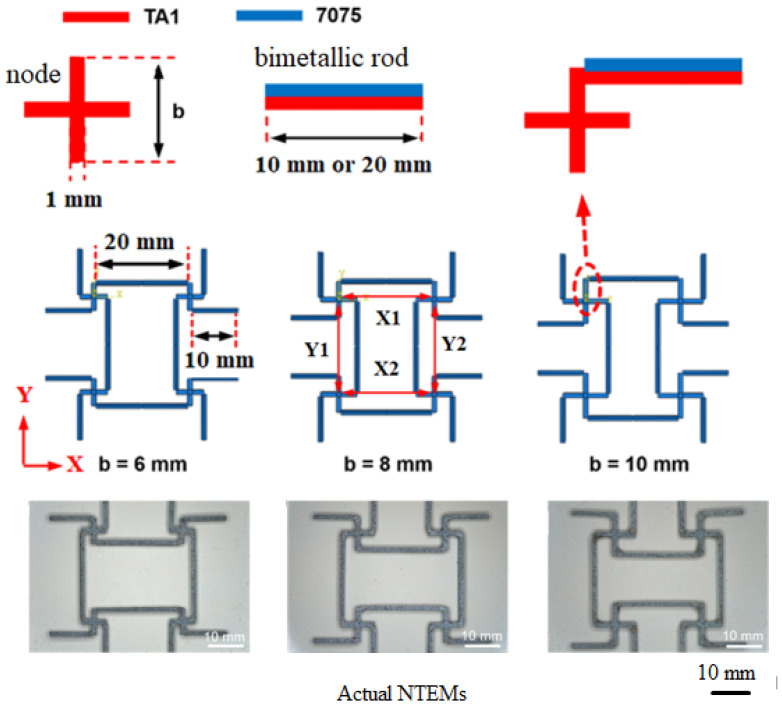
“Cross-shaped” node bending-dominated NTEMs.

The structures of the NTEMs are shown in Figure 2. The dimensions (mm) of the TA1 Ti node and Ti/Al rods were 1 × *b* (*b* = 6, 8, or 10) × 1 and 1.5 × 10 (or 20) × 1, respectively.

The nodes were bonded with the bimetallic rods using a high-temperature-resistant glue, and the assembled NTEMs are shown in Figure 2.

### 2.5. Optimization of the Thickness Ratio of Bimetallic Rods in “Cross-Shaped” Node Bending-Dominated NTEMs

**Figure 3:**
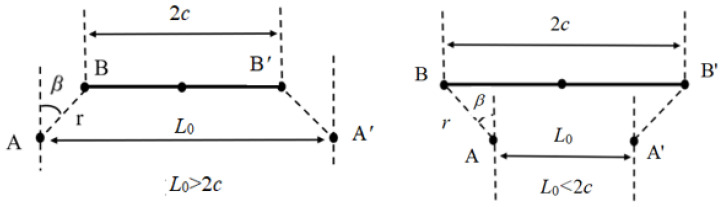
Two kinds of bending-type negative thermal expansion metamaterial [27].

There are many models for calculating *α* of NTEMs. The universal model of *α* for bending-type two-dimensional NTEMs with chiral/anti-chiral structures proposed in the literature [23] was selected to optimize the thickness ratio of bimetallic rods in “cross-shaped” node bending-dominated NTEMs. The nodes and rods in the NTEMs were simplified to points (A, B, ……) and lines (AB, ……), as shown in Figure 3.

The *α*_T_ for bending-type two-dimensional NTEMs with chiral/anti-chiral structures was [23]:

(1) $$\alpha_{T}=\left\{\begin{array}{c} {}[\frac{\rho \sin\frac{\theta }{2}-r\sin\left(\frac{\theta }{2}+\beta \right)}{c-r\sin\beta }-1]\frac{1}{\Delta T},\mathrm{anti}-\mathrm{chiral}\;\mathrm{with}\;L_{0}<2c\\ {}[\frac{\rho \sin\frac{\theta }{2}-r\sin\left(\frac{\theta }{2}-\beta \right)}{c+r\sin\beta }-1]\frac{1}{\Delta T},\;\mathrm{anti}-\mathrm{chiral}\;\mathrm{with}\;L_{0}>2c\\ {}[\frac{\sqrt{4\rho^{2}{sin}^{2}\frac{\theta }{2}-4\rho r\sin\frac{\theta }{2}\sin\left(\frac{\theta }{2}+\beta \right)+r^{2}}}{\sqrt{(c+r\sin\beta {)}^{2}+(r\cos\beta {)}^{2}}}-1]\frac{1}{\Delta T},\;\mathrm{chiral}\;\mathrm{with}\;L_{0}<2c\\ {}[\frac{\sqrt{4\rho^{2}{sin}^{2}\frac{\theta }{2}-4\rho r\sin\frac{\theta }{2}\sin\left(\frac{\theta }{2}-\beta \right)+r^{2}}}{\sqrt{(c-r\sin\beta {)}^{2}+(r\cos\beta {)}^{2}}}-1]\frac{1}{\Delta T},\mathrm{chiral}\;\mathrm{with}{\;L}_{0}>2c \end{array}\right.$$

where:

The angle between AB/A′B′ and BB′ was *β*. The lengths of AB, AA′, and BB′ were *r*, *L*_0_, and 2*c*, respectively. $\theta =\frac{l}{\rho },l=\left\{\begin{array}{c} 2c\left[1+\alpha_{1}\Delta T-\frac{\Delta \alpha \Delta T-\frac{t}{2\rho }}{1+\frac{1}{mn}}-\frac{t_{1}}{2\rho }\right]=2c\left[1+\alpha_{2}\Delta T+\frac{\Delta \alpha \Delta T-\frac{t}{2\rho }}{1+mn}+\frac{t_{2\rho }}{2}\right],\;\mathrm{anti}-\mathrm{chiral}\\ c\left[1+\alpha_{1}\Delta T-\frac{\Delta \alpha \Delta T-\frac{t}{2\rho }}{1+\frac{1}{mn}}-\frac{t_{1}}{2\rho }\right]=c\left[1+\alpha_{2}\Delta T+\frac{\Delta \alpha \Delta T-\frac{t}{2\rho }}{1+mn}+\frac{t_{2}}{2\rho }\right],\;\mathrm{chiral} \end{array}\right.$, $\rho =\frac{t\left[3{\left(1+m\right)}^{2}+\left(1+mn\right)\left(1+\frac{1}{mn}\right)\right]}{6{\left(1+m\right)}^{2}\Delta \alpha \Delta T}=\frac{t\left[3mn+1\right]}{6mn\Delta \alpha \Delta T}$, *m* = 1/(*t_1_*/*t*) − 1, *n* = *E*_2_/*E*_1_, and Δ*α* = *α*_1_ − *α*_2_. *E_i_*, *t_i_* and *α_i_* are the elastic modulus, thickness, and thermal expansion coefficients of material *i*, *i* = 1, 2; *t* = *t*_1_ + *t*_2_.

*t*_1_/*t* corresponding to the minimum *α*_T_ was defined as the optimal value.

### 2.6. Optimization Methods of Ti/Al Roll Bonding Process

**Figure 4:**
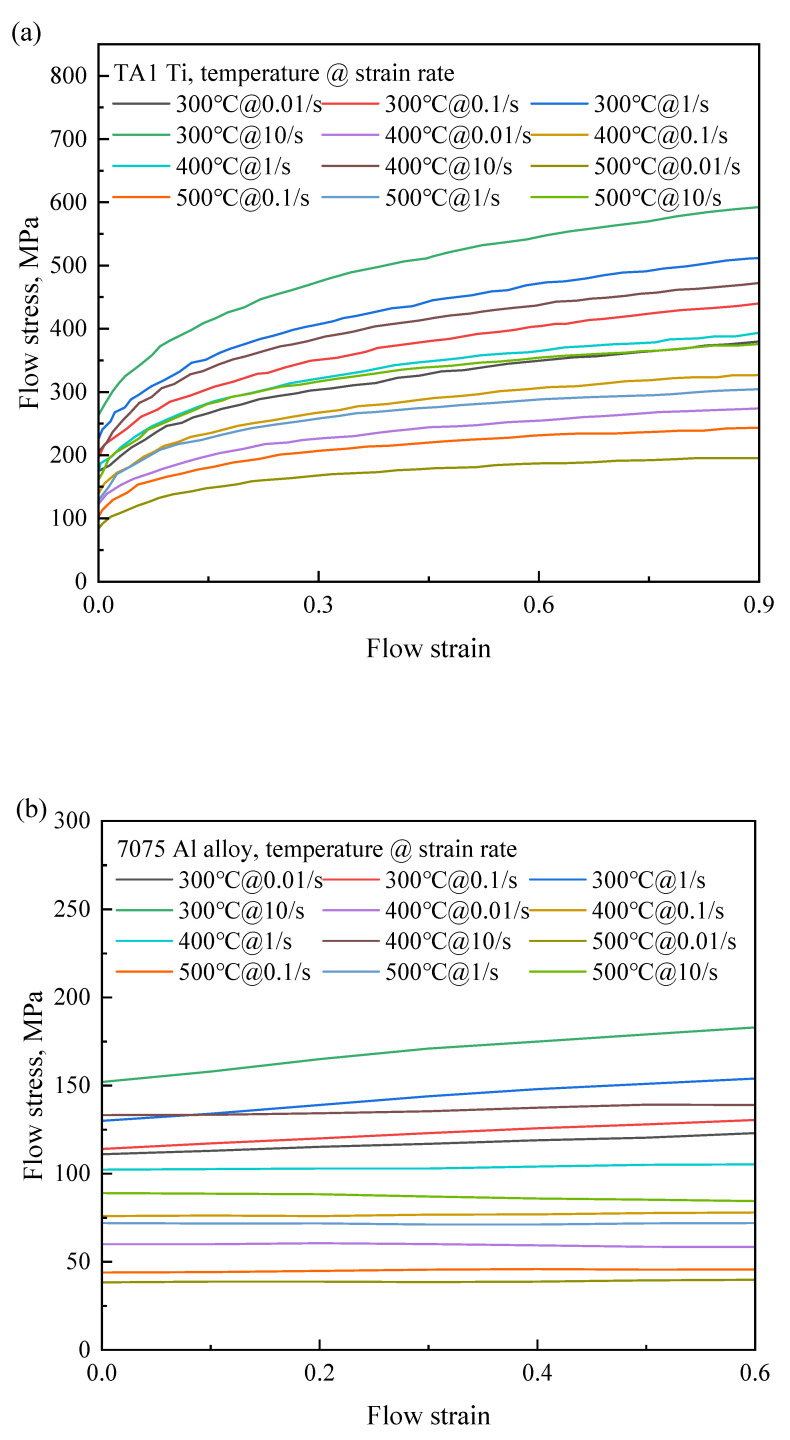
Flow stress vs. flow strain of TA1 and 7075 Al alloy. (**a**) TA1 Ti, and (**b**) 7075 Al alloy [data source: Material Library of the Simufact Forming 16].

Ti/Al roll bonding was optimized by FEM and verified using rolling experiments. The roll bonding process was simulated using the standard module of the finite element software Abaqus 2016. Rolls with a diameter of 200 mm were defined as rigid bodies. The billets (length = 50 mm) were defined as plastic bodies, and their flow stresses are shown in Figure 4. The friction coefficient was set to 0.8 in the simulation to ensure that the billets can be rolled into the rollers during the rolling process. CPE4RT type was selected for mesh generation. The parameters (rolling temperature, reduction ratio, and thickness of titanium and aluminum billets) corresponding to 0.55 < *t*_1_/*t* < 0.6 were defined as the optimal values.

## 3. Results

### 3.1. Optimization of the Thickness Ratio of Bimetallic

**Figure 5:**
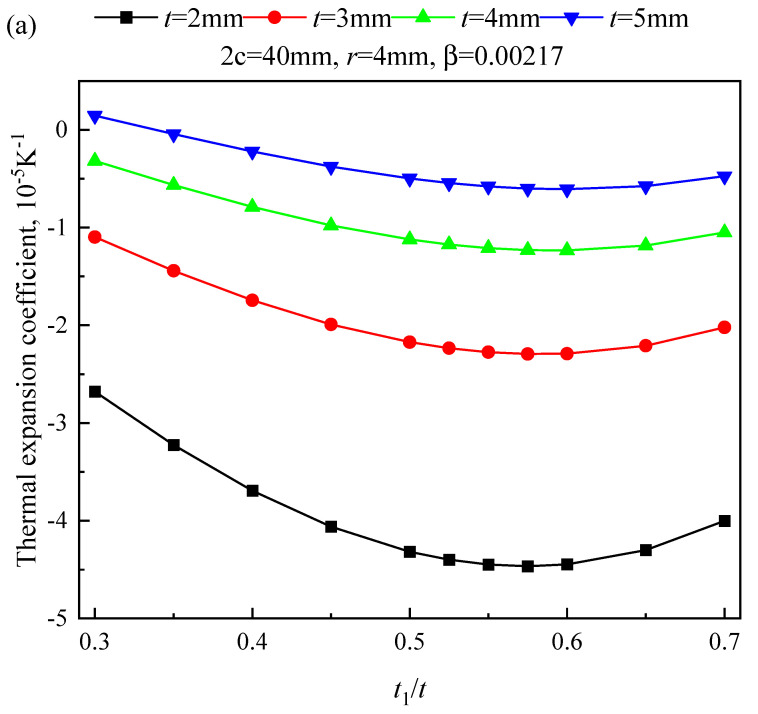

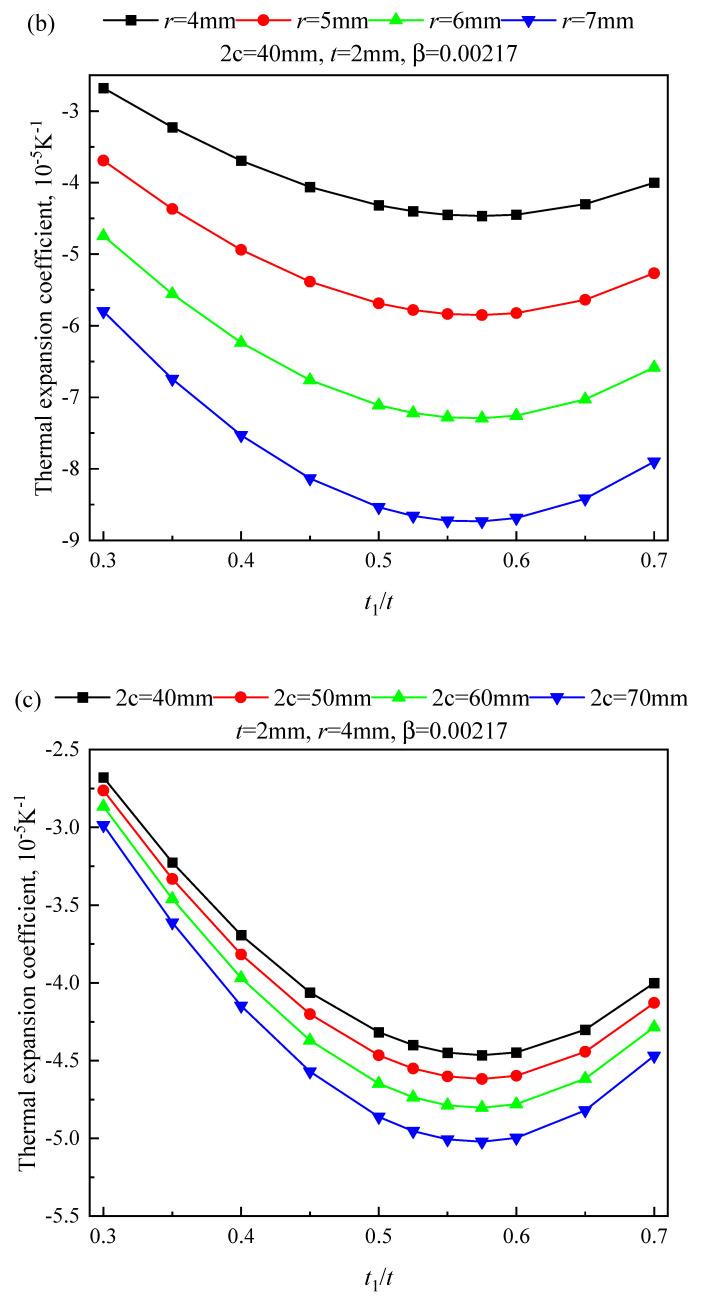
The αT of chiral/antichiral TA1 Ti/7075 Al NTEMs vs. *t*_1_/*t*. Effect of (**a**) t, (**b**) r, and (**c**) 2c.

According to Equation (1), α_T_ is affected by the elastic modulus ratio, thickness ratio, and *α* difference of the bimaterial rods. *E* and *α* of TA1 Ti were 110 GPa and 1.154 × 10^−5^/K, while those of the 7075 Al alloy were 70 GPa and 2.253 × 10^−5^/K, respectively. Therefore, the *α*_T_ values of TA1 Ti/7075 Al NTEMs were mainly affected by the thickness ratio of the Ti/Al rods. The *α*_T_ of the Ti/Al NTEMs increased and then decreased with a thickness ratio *t*_1_/*t* of 7075 Al alloy billet to the total billet. The peak value was obtained when 0.55 < *t*_1_/*t* < 0.6, as shown in Figure 5.

### 3.2. Optimization of Ti/Al Roll Bonding Process

**Figure 6:**
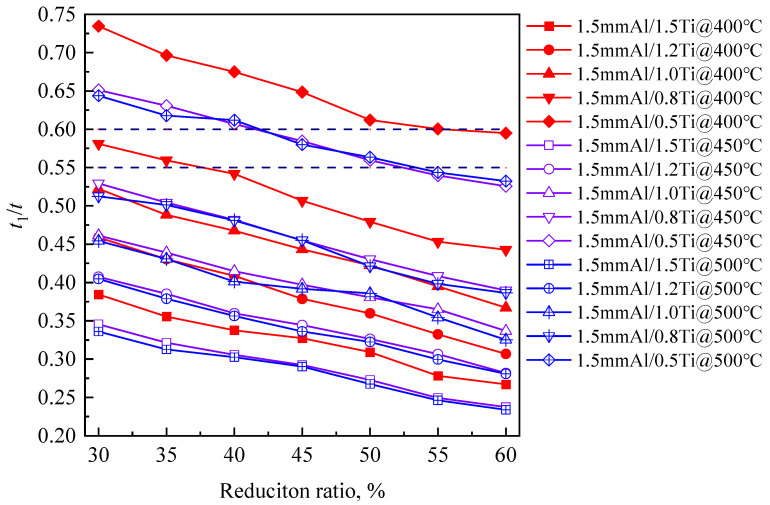
The simulated thickness ratio *t*_1_/*t* of 707 Al alloy billet to the total billet.

Variations in the simulated *t*_1_/*t* with the reduction ratio and thicknesses of billets are shown in Figure 6. Eight conditions satisfied the optimized *t*_1_/*t*: For 0.8 mm Ti/1.5 mm Al billets rolled at 300 °C with 30% and 35% reduction ratios, *t*_1_/*t* was 0.58 and 0.56; For 0.5 mm Ti/1.5 mm Al billets rolled at 300 °C with 55% and 60% reduction ratios, *t*_1_/*t* was 0.59; For 0.5 mm Ti/1.5 mm Al billets rolled at 400 °C with 45% and 50% reduction ratios, *t*_1_/*t* was 0.59 and 0.56; For 0.5 mm Ti/1.5 mm Al billets rolled at 500 °C with 45% and 50% reduction ratios, *t*_1_/*t* was 0.58 and 0.56.

Rolling experiments were conducted according to the above parameters. Ti and Al alloy plates were separated when the billets were rolled at 300 °C with 30% and 35% reduction ratios. The Ti plates fractured when the billets were rolled at 300 °C with 55% and 60% reduction ratios. When the billets were rolled at 500 °C, the 7075 Al plate fractured due to overburning. The plates remained bonded when the billets were rolled at 400 °C with 45% and 50% reduction ratios, and no fractures appeared. The experimental *t*_1_/*t* agreed with the simulated values, as shown in Table 2.

**Figure 7:**
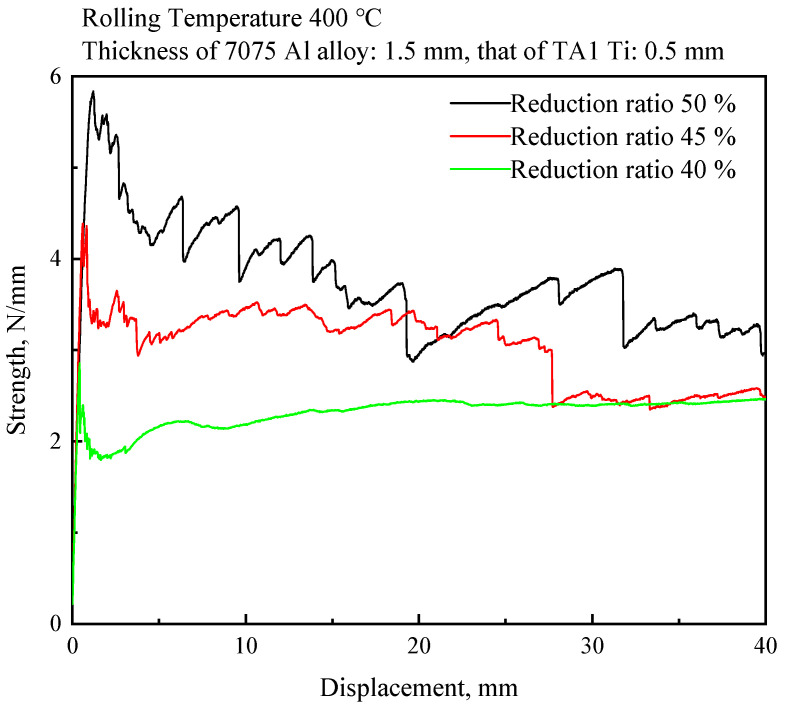
Peeling curve of rolled Ti/Al bimaterial rod.

The peeling test results are shown in Figure 7. For 0.5 mm Ti/1.5 mm Al rolled at 400 °C, the strengths of the bonding surfaces were 2.4 ± 0.52, 3.3 ± 0.43 and 4.2 ± 0.48 N/mm when the reduction ratios were 40%, 45%, and 50%, respectively.

### 3.3. Thermal Expansion Coefficients of the NTEMs

**Figure 8:**
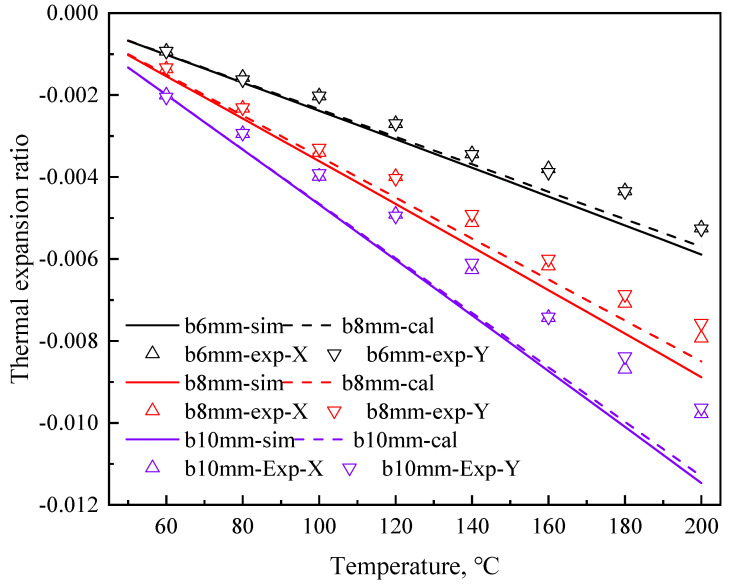
Thermal expansion ratio of the NTEMs.

The thermal expansion ratios of the NTEMs are shown in Figure 8. When *b* = 6 mm, 8 mm, and 10 mm, the *α*_T_ (K^–1^) values in the *X* and *Y* directions were –3.08 × 10^−5^ and –3.09 × 10^−5^; –4.66 × 10^−5^ and –4.45 × 10^−5^; –5.74 × 10^−5^ and –5.67 × 10^−5^, respectively.

## 4. Discussion

### 4.1. Bonding Strength of Ti/Al Bimaterial Rods for NTEMs

**Figure 9:**
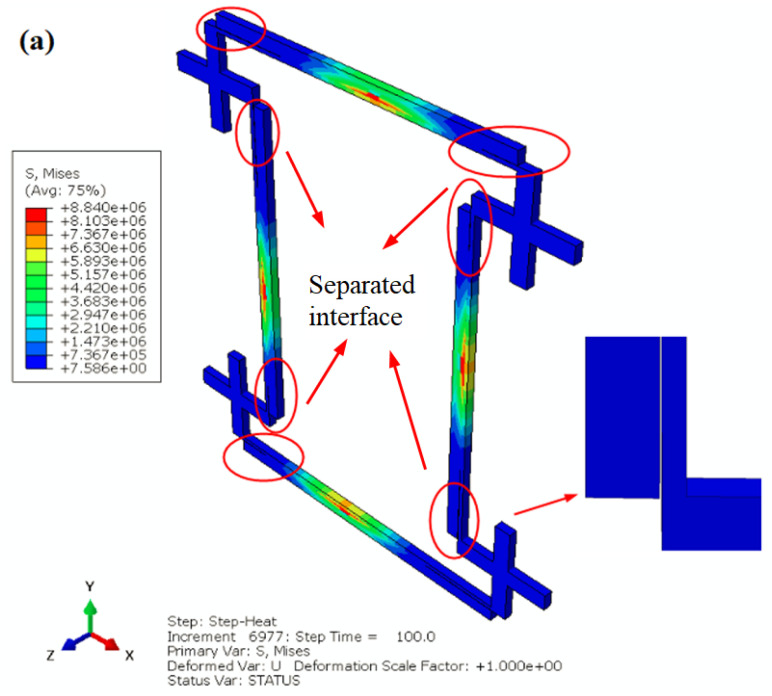

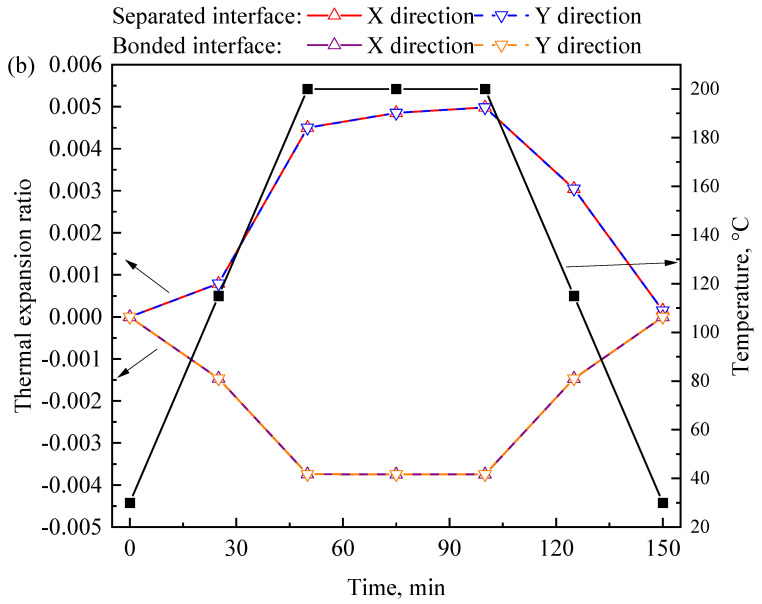
Separation of the bimetallic rods in NTEMs due to the thermal stress. (**a**) Separation of the bimetallic rods, and (**b**) thermal expansion ratio and temperature vs. time.

Thermal stresses exist at the interface of the bimetallic rods due to the different *α* values of the bimetallic rods. When the thermal stress exceeded the bonding strength of the bimetallic rods, the interface separated, and the *α* of the NTEMs shifted from negative to positive (Figure 9). Previous experiments have reported interfacial debonding due to a low bonding strength [29]. Therefore, improving the bonding strength of the rods should help ensure a stable *α* value of NTEMs.

Surface treatment of the billets, increasing the reduction ratio, and heat treatment are common methods to improve the bonding strength of rolled bimetallic plates. In this work, increasing the reduction ratio and heat treatment were used to improve the bonding strength of the rolled Ti/Al rods. The bonding strength of the Ti/Al rods rolled at a 50% reduction ratio was larger than those rolled at 45% and 40% (Figure 7). Therefore, the Ti/Al rods rolled at a 50% reduction ratio were further annealed at 400 °C, 450 °C, and 480 °C for 2 h and 4 h.

The bonding strengths (N/mm) of the Ti/Al rods annealed at 400 °C, 450 °C, and 480 °C for 2 h were 11.6 ± 0.68, 12.3 ± 0.86, and 10.1 ± 0.75, and those annealed for 4 h were 16.5 ± 0.59, 18.9 ± 0.64, and 15.5 ± 0.79, respectively. Annealing significantly improved the bonding strength of the rolled rods. The largest bonding strength of the rolled Ti/Al rods was 4.2 ± 0.48 N/mm, while that of the annealed ones was 18.9 ± 0.84 N/mm. These bonding strengths were larger than those of a Ti/Al 2-ply sheet rolled at 350 °C with a 38% reduction ratio and then annealed [31]. The bonding strength (N/mm) of the rolled Ti/Al 2-ply sheet was only 0.16 ± 0.04, while that of the rolled sheets annealed at 550 °C for 0.5, 1, 3, and 6 h were 8.77 ± 0.60, 9.28 ± 1.06, 11.33 ± 0.73, and 13.78 ± 0.45, respectively.

**Figure 10:**
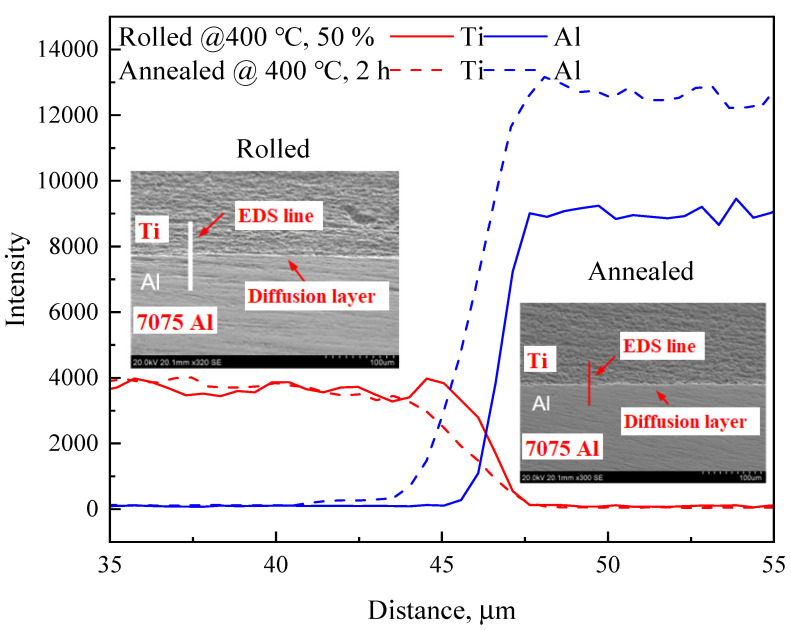
Elements distribution in the rolled and annealed TA1 Ti/7075 Al bimaterial rods.

Annealing improved the bonding strength of the rolled Ti/Al plates by increasing the diffusion layer thickness because larger regions become available for metallurgical bonding upon increasing atomic diffusion [31]. The morphology and elemental distribution of the interface of the samples are shown in Figure 10. A diffusion layer existed at the interface, but the diffusion layer width in the annealed sample was greater than in the rolled one. For example, the diffusion layer widths were 2.59, 3.18, and 3.67 µm when the samples were rolled at 400 °C with 40%, 45%, and 50% reduction ratios, respectively. The width increased to 4.92 µm or 7.25 µm after the samples were annealed at 400 °C for 2 h or 480 °C for 4 h. In addition, there was no stepwise change in the elemental distribution of the diffusion layer, which indicated no intermetallic compounds were formed.

### 4.2. Isotropic Negative Thermal Expansion Properties of the TA1 Ti/7075 Al NTEMs

To quantitatively assess the isotropy of the thermal expansion properties, the isotropy ratio *IR* was used, which is defined as:

(2) $$IR=1-\left|\frac{2\bar{\alpha_{X}}}{\bar{\alpha_{X}}+\bar{\alpha_{Y}}}-1\right|=1-\left|\frac{2\bar{\alpha_{Y}}}{\bar{\alpha_{X}}+\bar{\alpha_{Y}}}-1\right|=1-\left|\frac{2\bar{\varepsilon_{X}}}{\bar{\varepsilon_{X}}+\bar{\varepsilon_{Y}}}-1\right|=1-\left|\frac{2\bar{\varepsilon_{Y}}}{\bar{\varepsilon_{X}}+\bar{\varepsilon_{Y}}}-1\right|$$

where $\bar{\alpha_{X}}$ and $\bar{\alpha_{Y}}$, $\bar{\varepsilon_{X}}$ and $\bar{\varepsilon_{Y}}$ are the average thermal expansion coefficients and thermal expansion ratios along two orthogonal directions (*X* and *Y*), respectively.

*IR* ranges from 0 to 1, where a value closer to 1 indicates greater isotropy. The anisotropy ratio *AR* was defined as 1−*IR*, which ranged from 0 to 1, where a value closer to 1 indicates greater anisotropy.

**Figure 11:**
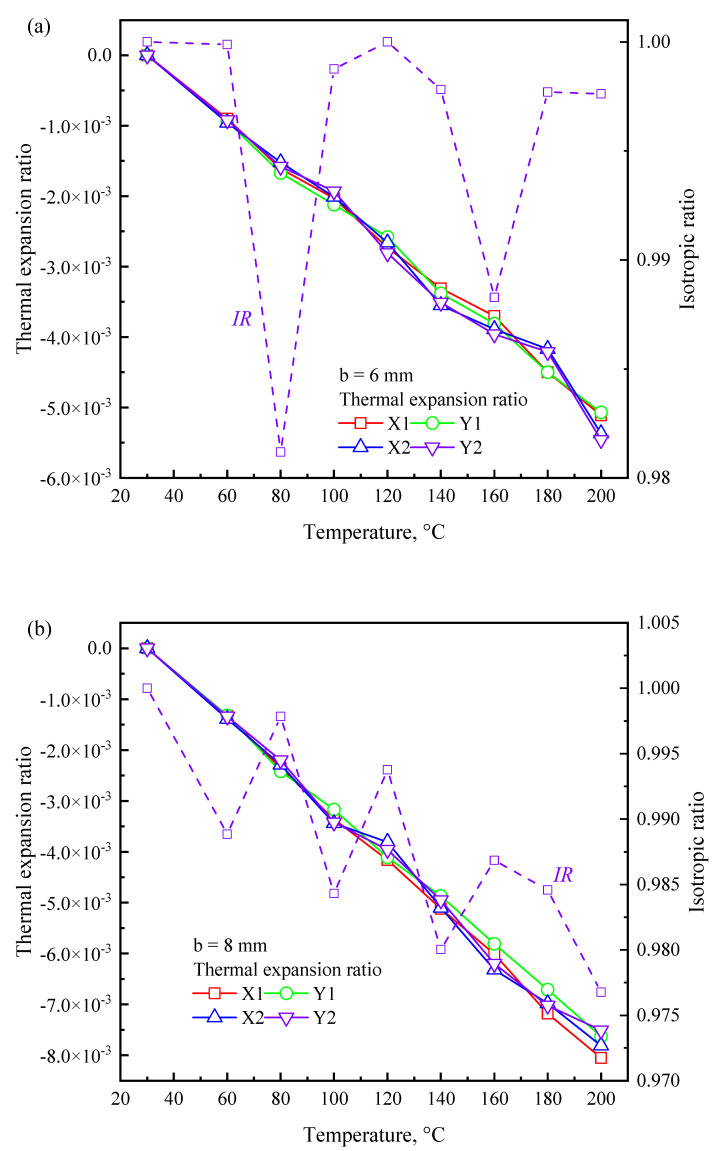

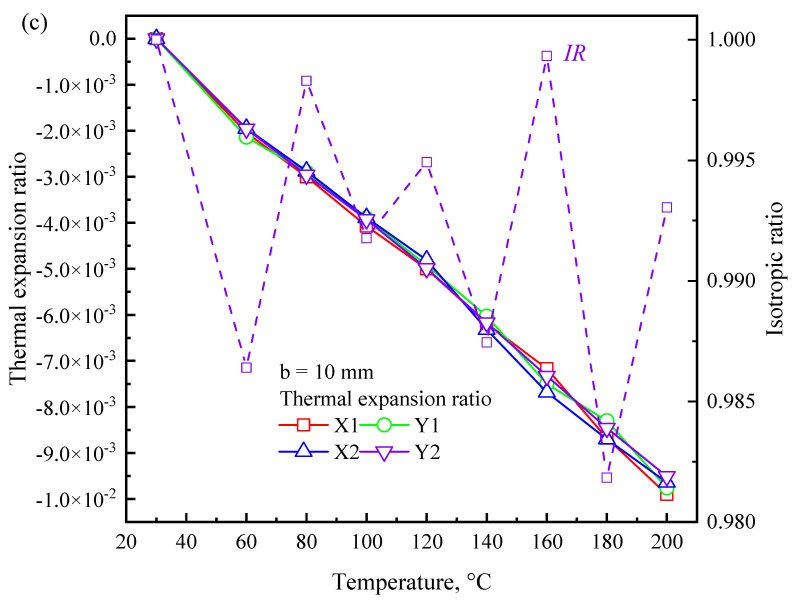
Isotropic negative thermal expansion properties of the TA1 Ti/7075 Al NTEMs. b was (**a**) 6 mm, (**b**) 8 mm, and (**c**) 10 mm.

The *IR* values in refs. [9,31] calculated according to Equation (2) were 0.977 and 0.987, respectively. The *IR* of the Ti/Al NTEMs were >0.975, as shown in Figure 11 (X1, X2, Y1, and Y2 are shown in Figure 1), indicating that the NTEs of the Ti/Al NTEMs were isotropic.

To prove the above conclusions, a *t*-test (two samples assuming unequal variance) was performed on the thermal expansion ratio of the NTEMs, and the results are shown in Table 3. Parameter *P* indicates the significance of a parameter (in this work, the parameter was the direction, *X* or *Y*), where 0 ≤ *P* ≤ 0.01 indicates highly significant; 0.01 < P ≤ 0.05 indicates significant; 0.05 < *P* ≤0.20 indicates weakly significant; 0.20 < *P* ≤ 1 indicates not significant. Therefore, 0 ≤ *P* ≤ 0.05 indicates that the thermal expansions in the *X* and *Y* directions of the NTEMs were very different (anisotropy) and vice versa (isotropy). The *p* values (single and double-tailed) in Table 3 were greater than 0.2, indicating that NTEMs underwent isotropic thermal expansion.

### 4.3. Cyclic Thermal Stability of Ti/Al NTEMs

**Figure 12:**
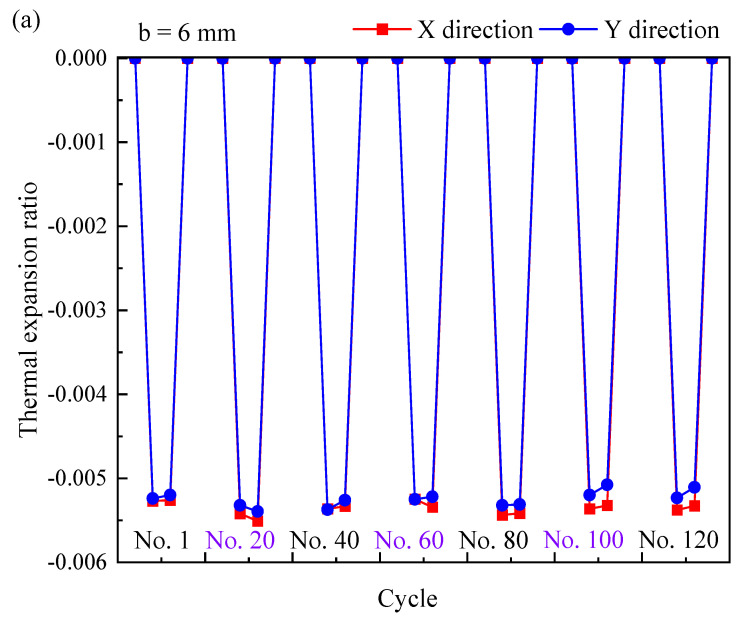

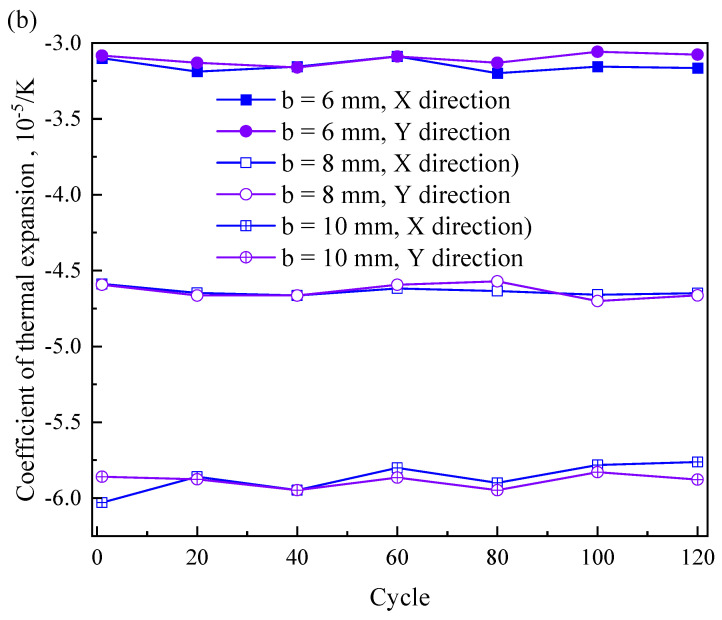
Thermal expansion properties of the TA1 Ti/7075 Al NTEMs during thermal cycling. (**a**) Thermal expansion ratios vs. cycle, and (**b**) α vs. cycle.

The typical expansion ratios and *α* values of the NTEMs under a cyclic thermal load are shown in Figure 12a,b. The cyclic thermal stability ratio *SR* was defined to characterize the cyclic thermal stability of the thermal expansion:

(3) $$SR=\left|\frac{\alpha_{i=J}}{\alpha_{i=1}}-1\right|=\left|\frac{\varepsilon_{i=J}}{\varepsilon_{i=1}}-1\right|$$

where *i* = 1 indicates cycle 1, and *i* = *J* represents cycle *J*.

An *SR* value closer to 0 indicates greater cyclic stability. The *SR*s of the Ti/Al NTEMs are listed in Table 4, which were all less than 0.05, indicating their good cyclic stability during thermal expansion. The *IR*s of Ti/Al NTEMs during thermal cycles are listed in Table 4, in which *IR* > 0.98, further demonstrating the good thermal cyclic stability of the NTEMs.

The cyclic stability of the NTEMs was further verified by probabilistic statistical methods. The *SR* and *IR* values of the NTEMs were analyzed by unrepeated two-factor ANOVA, and the results are shown in Table 5. The two factors were the cycle number and length of the cross-shaped node *b*. The *p*-values of the cycle were 0.73, 0.11, and 0.34 for *SR* along the *X* and *Y* directions and *IR*. This analysis indicated that the NTEMs showed excellent thermal stability.

After thermal cycling experiments, no separation of Ti and Al plates was found in the Ti/Al NTEMs, indicating that roll-bonding and annealing ensured sufficient interfacial strength.

### 4.4. Errors Between the Modeled and Experimental Results of the αT of the TA1 Ti/7075 Al NTEMs

**Figure 13:**
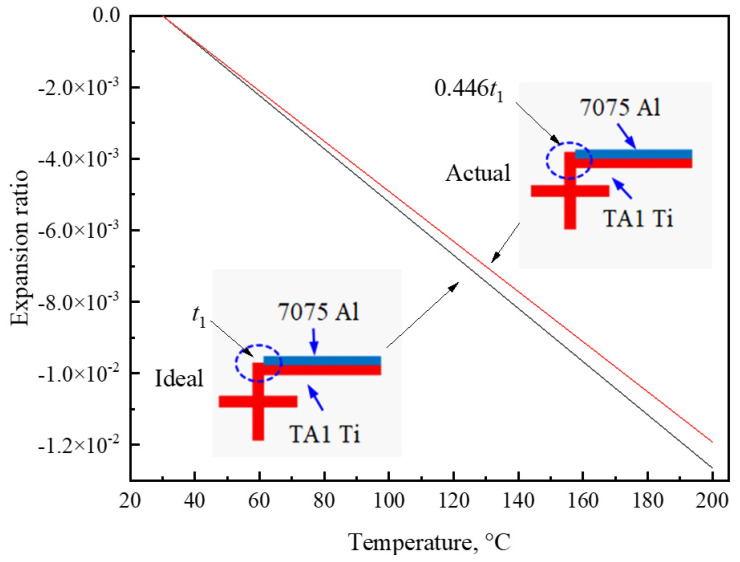
Effect of structural mismatch on the thermal expansion of TA1 Ti/7075 Al NTEMs.

As shown in Figure 8, the experimental *α*_T_ values were less than the calculated values. When *b* = 6 mm, 8 mm, and 10 mm, the relative errors ranged from 3.07% to 16.3%, from 2.94% to 12.8%, and from 0.5% to 21.8%, respectively. The main reasons for the difference included mismatches between the rods and nodes due to manual operation, and the high-temperature oxidation of the metals during heating. As shown by the ideal case in Figure 13, the rods glued to the nodes in the NTEM should be TA1 Ti rods. The thickness of the TA1 Ti layer in the rolled rods was less than 0.5 mm, and some of the Al rod was easily glued to the node, i.e., mismatch occurred, as shown by the actual case in Figure 13. Equation (1) did not provide an accurate *α*_T_ value for this structure due to mismatch. The results of Equation (1) agreed with the FEM results (Figure 8). Therefore, the FEM was used instead of Equation (1) to study the effect of mismatch on the *α*_T_ of NTEMs, and the results are shown in Figure 13. The mismatch lowered the *α*_T_ value of the Ti/Al NTEMs, resulting in a discrepancy between the experimental results and the model predictions.

Experiments were carried out in the air, and oxidation occurred in the metals at elevated temperatures. The *α* value of metal oxides is usually lower than that of the corresponding metals. For example, the *α* of Ti and TiO_2_ were 11.5 × 10^−6^/K and 7.14 × 10^−6^/K, respectively, while those of Al and Al_2_O_3_ were 22.5 × 10^−6^/K and 7.5 × 10^−6^/K, respectively. This also affected the thermal expansion of the NTEMs.

## 5. Conclusions

(1) A universal model describing the thermal expansion coefficient of bending-type two-dimensional metamaterials with chiral/anti-chiral structures proposed was employed to optimize the thickness ratio of bimetallic rods in NTEMs. For cross-type NTEMs consisting of TA1 Ti/7075 Al bimetallic rods, a relatively high thermal expansion coefficient was obtained when the thickness ratio of the 7075 Al alloy to the rods was in the range of 0.56–0.60.

(2) The optimized roll bonding process to meet this thickness ratio was: a rolling temperature of 400 °C, a reduction ratio of 50%, and TA1 Ti and 7075 Al billet thicknesses of 0.5 mm and 1.5 mm, respectively.

(3) The rolled Ti/Al rods had a maximum bonding strength of 18.9 N/mm after being annealed at 450 °C for 4 h. NTEMs were prepared using annealed Ti/Al bimaterial rods and Ti nodes.

(4) The isotropy ratio *IR* and cyclic thermal stability ratio *SR* were proposed to quantitatively assess the isotropy and thermal stability of the thermal expansion properties of NTEMs. The results showed that the thermal expansion of the Ti/Al NTEMs was isotropic and thermally stable, as verified by probabilistic statistical methods.

## Figures and Tables

**Table 1:** Chemical composition (wt.%) of TA1 pure Ti and 7075 Al alloy.

Material	H	C	O	Fe	Ti	Cr	Mn	Si	Cu	Mg	Zn	Al
TA1	0.014	0.08	0.15	0.03	Bal.	-	-	-	-	-	-	-
7075	-	-	-	0.5	0.2	0.23	0.3	0.4	1.6	2.5	5.6	Bal.

**Table 2:** Experimental and simulated *t*_1_/*t* of rolled bimaterial rods. Rolling temperature was 400 °C.

	Reduction ratio, %		
	40	45	50
Simulated	0.61	0.59	0.56
Experimental	0.62	0.58	0.55

**Table 3:** Results of *t*-test (two samples assuming unequal variance) for thermal expansion ratio of TA1 Ti/7075 Al NTEMs along the X and Y directions.

Data source:						
Temperature, °C	b = 6 mm		b = 8 mm		b = 10 mm	
	X	Y	X	Y	X	Y
60	−0.0009	−0.0009	−0.0013	−0.0013	−0.002	−0.0020
80	−0.0015	−0.0016	−0.0023	−0.0023	−0.0029	−0.0029
100	−0.0020	−0.0020	−0.0034	−0.0033	−0.0039	−0.0039
120	−0.0027	−0.0027	−0.0039	−0.0040	−0.0049	−0.0049
140	−0.0034	−0.0034	−0.0051	−0.0049	−0.0062	−0.0061
160	−0.0038	−0.0038	−0.0061	−0.0060	−0.0074	−0.0074
180	−0.0043	−0.0043	−0.0070	−0.0068	−0.0086	−0.0083
200	−0.0052	−0.0052	−0.0079	−0.0075	−0.0097	−0.0096
*t*-Test: two samples assuming unequal variance						
	Variable 1	Variable 2	Variable 1	Variable 2	Variable 1	Variable 2
Mean	−0.00267	−0.00269	−0.0041	−0.0040	−0.0051	−0.0050
Variance	2.88 × 10^−6^	2.91 × 10^−6^	7.1 × 10^−6^	6.53 × 10^−6^	1.04 × 10^−6^	9.89 × 10^−6^
Observations	9	9	9	9	9	9
Pooled Variance	0		0		0	
df	16		16		16	
t Stat	0.02974		−0.0929		−0.0429	
P(T ≤t) one-tail	0.48831		0.46353		0.48313	
t Critical one-tail	1.74588		1.74588		1.74588	
P(T ≤t) two-tail	0.97663		0.92707		0.96627	
t Critical one-tail	2.11990		2.11990		2.11990	

**Table 4:** SR and IR of the Ti/Al NTEMs.

Cycle	*SR* for X Direction			*SR* for X Direction			*IR*		
	b, mm			b, mm			b, mm		
	6	8	10	6	8	10	6	8	10
1	-	-	-	-	-	-	0.997	0.999	0.986
20	0.0284	0.0128	0.0282	0.0156	0.0154	0.0029	0.991	0.998	0.999
40	0.0178	0.0167	0.0136	0.0254	0.0154	0.0150	0.999	1	1
60	0.0039	0.0065	0.0379	0.0019	0	0.0010	1	0.997	0.994
80	0.0318	0.0102	0.0214	0.0156	0.0050	0.0150	0.989	0.993	0.996
100	0.0178	0.0154	0.0409	0.0078	0.0230	0.0051	0.984	0.996	0.996
120	0.0208	0.0133	0.0442	0.0019	0.0152	0.0032	0.986	0.998	0.99

**Table 5:** Unrepeated two-factor ANOVA results.

Source	SS	df	MS	F	*p*-Value	F Crit
SR along X direction						
Cycle	0.00027	5	5.56 × 10^−5^	0.55873	0.72974	3.3258
b	0.00104	2	0.000521	5.23555	0.02781	4.1028
Error	0.00099	10	9.95 × 10^−5^			
SR along Y direction						
Cycle	0.00052	5	0.000105	2.3410	0.1182	3.3258
b	9.63 × 10^−5^	2	4.82 × 10^−5^	1.0744	0.3778	4.1028
Error	0.00044	10	4.48 × 10^−5^			
*IR*						
Cycle	0.00067	5	0.00011	1.2599	0.3442	2.9961
b	0.00037	2	0.00018	2.0784	0.1678	3.8852
Error	0.00107	10	8.98 × 10^−5^			

## Data Availability

All data generated or analyzed during this study are included in this article.
